# Dengue Outbreak and Response — Puerto Rico, 2024

**DOI:** 10.15585/mmwr.mm7405a1

**Published:** 2025-02-20

**Authors:** Fhallon Ware-Gilmore, Dania M. Rodriguez, Kyle Ryff, MPH, Jomil M. Torres, Miladys Perez Velez, Cristhian T. Torres-Toro, Gilberto A. Santiago, Aidsa Rivera, Zachary J. Madewell, Yashira Maldonado, Iris Cardona-Gerena, Grayson C. Brown, Laura E. Adams, Gabriela Paz-Bailey, Melissa Marzán-Rodriguez

**Affiliations:** ^1^Epidemic Intelligence Service, CDC; ^2^Division of Vector-Borne Diseases, National Center for Emerging and Zoonotic Infectious Diseases, CDC, Puerto Rico; ^3^Career Epidemiology Field Officer Program, CDC; ^4^Puerto Rico Department of Health; ^5^Puerto Rico Vector Control Unit.

**Summary**

**What is already known about this topic?**

Dengue, a mosquitoborne disease that can lead to severe illness or death, is endemic in tropical and subtropical regions worldwide. The most recent outbreak in Puerto Rico occurred in 2013.

**What is added by this report?**

During 2024, Puerto Rico reported 6,291 dengue cases and surpassed the epidemic threshold, prompting declaration of a local public health emergency. Approximately one half of patients (52.3%) were hospitalized, 264 (4.2%) had severe dengue cases, and 11 (0.2%) persons died. Persons aged 10–19 years accounted for 28.4% of severe cases.

**What are the implications for public health practice?**

Improved case recognition and clinical management facilitate improved outcomes. To reduce mosquito bite risk, residents of and visitors to Puerto Rico should consider using repellents, wearing protective clothing, and staying in places with door and window screens.

## Abstract

Dengue, a mosquitoborne viral infection, is a public health threat in Puerto Rico, where multiple dengue virus (DENV) serotypes circulate. Dengue causes an acute febrile illness that can progress to severe disease or death. The last outbreak declared by the Puerto Rico Department of Health occurred during 2013. In January 2024, the number of dengue cases in Puerto Rico surpassed the epidemic threshold and remained elevated, prompting the Puerto Rico Department of Health to declare a public health emergency in March 2024. In collaboration with CDC, a dengue outbreak response was initiated to monitor the outbreak and implement vector-control measures alongside public health campaigns to raise awareness about increasing dengue case numbers and strategies to prevent mosquito bites. During 2024, a total of 6,291 confirmed dengue cases were reported; the highest numbers of cases were reported in the municipalities of San Juan (1,200; 17.3%), Carolina (354; 5.1%), and Rincón (252; 3.6%). DENV serotype 3 predominated, accounting for 59.2% of cases with known serotype. Approximately one half of ill patients (52.3%) required hospitalization, with the highest percentages of hospitalizations (33.9%) and severe dengue cases (28.4%) occurring among persons aged 10–19 years. Overall, severe dengue was identified in 4.2% of cases, with 11 reported fatalities (0.2%). Transmission remains elevated in multiple regions, underscoring the need for tailored public health measures, including vaccination among eligible populations, vector management, community outreach, and provider education to facilitate improved outcomes. To reduce the risk for mosquito bites, residents of and visitors to Puerto Rico should consider using repellents, wearing protective clothing, and staying in places with door and window screens.

## Introduction

Dengue is a mosquitoborne viral infection primarily transmitted by *Aedes aegypti* mosquitoes. Dengue is caused by four distinct dengue virus (DENV) serotypes (DENV-1–4), and manifestations range from asymptomatic infection to severe disease (*1*). Signs and symptoms typically appear 3–10 days after exposure and include fever, muscle and joint pain, retroorbital pain, rash, nausea, and vomiting. Although no antiviral treatment is available, early recognition of warning signs of severe dengue, a life-threatening complication of dengue, proper triage, supportive care, and follow-up are crucial to reducing morbidity and mortality (*2*). The Dengvaxia dengue vaccine (Sanofi-Pasteur)* is recommended for persons aged 9–16 years who live in areas of the United States with endemic dengue and who have had a confirmed previous dengue infection; vaccination provides protection against symptomatic disease and hospitalization from dengue.^†^

Approximately 13 million dengue cases were reported to the Pan American Health Organization from North, Central, and South America in 2024 (*3*). DENV transmission is frequent in Puerto Rico, with seasonal peaks occurring July–November and outbreaks every 3–7 years (*4*). The dengue outbreak in 2024 marks Puerto Rico's first since 2013, denoting an unusually long 11-year gap. This extended interval was likely influenced by temporary cross-protective immunity to dengue after the Zika outbreak in 2016 followed by reduced travel and exposure during the COVID-19 pandemic.

In March 2024, the Puerto Rico Department of Health (PRDH) declared a public health emergency when dengue cases exceeded the epidemic threshold, defined as the point at which weekly cases surpass levels historically associated with epidemics, based on 30 years of surveillance data (*5*,*6*). This report describes a dengue outbreak response conducted in collaboration with CDC to monitor the outbreak and implement vector-control measures and public health campaigns to raise awareness about increasing dengue case numbers and strategies to prevent mosquito bites.

## Investigation and Findings

### Data Sources and Analysis

Dengue cases were classified based on the Council of State and Territorial Epidemiologists case definition (*7*). Serum specimens from persons suspected to have dengue were tested using reverse transcription–polymerase chain reaction (RT-PCR) for DENV and by enzyme-linked immunosorbent assay for the presence of nonstructural protein 1 (NS1) or immunoglobulin M (IgM); positive test results from commercial laboratories were reported to PRDH. To enhance case tracking and reporting, PRDH upgraded the existing arboviral case investigation system to a new platform that integrates data streams from commercial laboratories. Disease incidence (dengue cases per 100,000 population, using 2020 U.S. Census Bureau data) was assessed using temporal trends, geographic distribution in high-incidence areas, age- and serotype-specific incidence, and evaluations of hospitalizations and severe outcomes. This activity was reviewed by CDC, deemed not research, and was conducted consistent with applicable federal law and CDC policy.^§^

### Epidemiologic Surveillance

**FIGURE 1:**
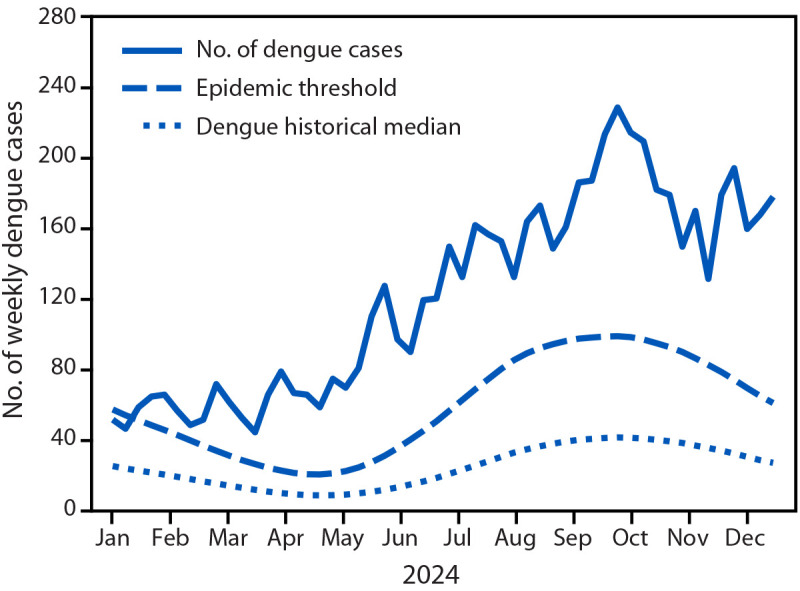
Number of weekly dengue cases, historical median,* and the epidemic threshold^†^ — Puerto Rico, 2024 * The median weekly number of cases since 1986. ^†^ The weekly dengue epidemic threshold is defined as the 75th percentile of a negative binomial regression model fit to ≥30 years of historical, probable, and confirmed dengue case data in Puerto Rico. This 75th percentile threshold aligns best with historical epidemic classifications, and roughly corresponds to expected epidemics every 4 years.

In 2024, a total of 6,291 dengue cases (191.4 per 100,000 population) were reported in Puerto Rico. Cases remained above the epidemic threshold throughout 2024 and were particularly high after the start of the rainy season in May (Figure 1). The highest number of cases was reported in October (884); the peak number of weekly cases reached 226 during week 40 (October). Active transmission remained above the epidemic threshold through January 2025.

**FIGURE 2:**
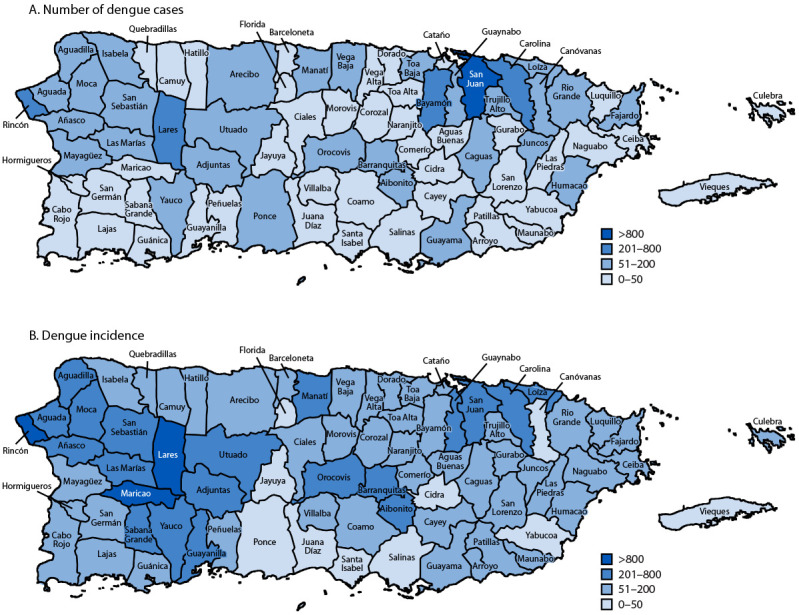
Number of dengue cases (A) and dengue incidence per 100,000 population* (B), by municipality — Puerto Rico, 2024 * Based on 2020 U.S. Census Bureau data.

Although dengue cases were reported across the island, the highest case counts were concentrated in the San Juan metropolitan area: 1,200 cases (17.3%) were reported in San Juan, followed by Carolina (254; 5.1%), Rincón (252; 3.6%), Lares (240; 3.5%), and Bayamón (233; 3.4%) (Figure 2). The highest incidences were reported in Rincón (1,659.3 per 100,000), followed by Maricao (925.3), Lares (853.9), and Orocovis (765.1).

### Demographic Characteristics of Dengue Patients

Of the 6,291 total cases, 3,364 (53.5%) were among males (Table). Persons aged 10–19 years accounted for the largest number and percentage of cases (1,845; 29.3%), followed by adults aged 20–29 years (1,034; 16.4%). The median patient age was 24 years (range = 2.3 months–99 years). 

**TABLE:** Characteristics of dengue cases — Puerto Rico, 2024

Characteristic	No. (col. %)						
	Total dengue cases		Hospitalized dengue patients		Severe dengue* cases		Fatal dengue cases
**Total**	**6,291 (100)**		**3,289 (52.3)**		**264 (4.2)**		**11 (0.2)**
**Age group, yrs**							
<1	**26 (0.4)**		20 (0.6)		0 (—)		0 (—)
1–9	**518 (8.2)**		242 (7.4)		11 (4.2)		0 (—)
10–19	**1,845 (29.3)**		1,115 (33.9)		75 (28.4)		1 (9.1)
20–29	**1,034 (16.4)**		492 (15.0)		54 (20.5)		1 (9.1)
30–39	**766 (12.2)**		348 (10.6)		38 (14.4)		1 (9.1)
40–49	**643 (10.2)**		306 (9.3)		22 (8.3)		0 (—)
50–59	**585 (9.3)**		276 (8.4)		20 (7.6)		2 (18.2)
60–69	**454 (7.2)**		253 (7.7)		27 (10.2)		2 (18.2)
≥70	**420 (6.7)**		237 (7.2)		17 (6.4)		4 (36.4)
**Sex**							
Female	**2,926 (46.5)**		1,505 (45.8)		123 (46.6)		4 (36.4)
Male	**3,364 (53.5)**		1,784 (54.2)		141 (53.4)		7 (63.6)
Unknown	**1 (<1)**		0 (—)		0 (—)		0 (—)
**Laboratory testing **							
RT-PCR positive	**4,942 (78.6)**	2,910 (88.5)		233 (88.3)		8 (72.2)	
NS1 positive-only	**293 (4.7)**	105 (3.2)		7 (2.7)		0 (—)	
IgM positive-only	**1,056 (16.8)**	274 (8.3)		24 (9.1)		3 (27.3)	
**DENV serotype among RT-PCR–positive cases (n = 4,942)**							
DENV-1	**1,367 (27.7)**		766 (26.3)		73 (31.3)		0 (—)
DENV-2	**636 (12.9)**		433 (14.9)		40 (17.2)		1 (12.5)
DENV-3	**2,926 (59.2)**		1,709 (58.7)		120 (51.5)		7 (87.5)
DENV-4	**1 (<1)**		0 (—)		0 (—)		0 (—)
Serotype unavailable	**12 (0.2)**		2 (0.1)		0 (—)		0 (—)

**Abbreviations**: DENV = dengue virus; IgM = immunoglobulin M; NS1 = nonstructural protein 1; RT-PCR = reverse transcription–polymerase chain reaction.

* https://ndc.services.cdc.gov/case-definitions/dengue-virus-infections-2015

### Illness Characteristics and Outcomes

Among the 3,289 (52.3%) dengue patients who required hospitalization, the highest number and percentage occurred among persons aged 10–19 years (1,115 hospitalizations; 33.9%), followed by adults aged 20–29 years (492; 15.0%). Severe dengue, a condition characterized by life-threatening complications including shock, bleeding, and organ failure, was confirmed in 264 patients (4.2% of total cases), with the highest number and percentage among persons aged 10–19 years (75; 28.4%), followed by adults aged 20–29 years (54; 20.5%). Eleven deaths were reported (0.2% of total cases), with the highest number among persons aged ≥70 years (four deaths; 36.4%), followed by two deaths each among persons aged 50–59 years and 60–69 years (18.2% each).

### Virologic Surveillance

Virologic surveillance identified DENV-3 as the predominant circulating serotype, accounting for 2,926 (59.2%) of all reported cases positive by RT-PCR (4,942 cases), followed by DENV-1 (1,367; 27.7%). Phylogenetic analyses found that the three serotypes circulating during the outbreak (DENV-1, DENV-2, and DENV-3) were different variants from those previously present in Puerto Rico and had been introduced to the island during 2019–2023.

## Public Health Response

### Enhancing Surveillance

To increase outbreak awareness and improve dengue case recognition among health care providers, PRDH and CDC have conducted 3,030 education and prevention events, reaching approximately 167,000 persons across all eight health regions. These efforts include health fairs, virtual conferences, and community outreach, supported by municipal epidemiologists, central-level educators, and community outreach specialists.

 In October 2023, PRDH developed a mandatory dengue training course for medical professionals to raise awareness of dengue and prepare for future outbreaks on the island. Efforts to implement and expand training intensified during the 2024 outbreak, and by November, approximately 10,800 health care providers completed the course through in-person sessions and webinars. 

To improve case tracking and reporting, PRDH upgraded the existing arboviral case investigation system to a new, comprehensive database, improving data accuracy and management. CDC and PRDH collaborated to expand dengue testing capacity by providing guidance to commercial laboratories, enabling broader access for patients and more rapid diagnostic testing by working with existing specimen transport and reporting systems at commercial laboratories. In addition, PRDH established a free dengue testing center for patients with a medical order, offering an acute dengue panel with RT-PCR and IgM testing. PRDH also intensified surveillance activities by conducting interviews with all persons with confirmed dengue to collect comprehensive case data, including detailed exposure history and symptom progression. PRDH conducted weekly coordination meetings with CDC, the Puerto Rico Vector Control Unit (PRVCU), and other partners to review trends, update protocols, coordinate messaging and outreach, direct vector control efforts, and refine overall response strategies.

### Mosquito Surveillance and Control Activities

To support mosquito surveillance and control activities, PRVCU and PRDH Environmental Health staff members collaborated with PRDH epidemiologists to perform mosquito trapping. During January–November 2024, PRVCU captured 234,713 *A. aegypti* mosquitoes across Puerto Rico using autocidal gravid ovitraps. A total of 28,932 mosquito pools were tested using RT-PCR, which identified 316 (1.1%) positive pools. The highest number of positive DENV detections in mosquito pools was recorded in San Juan (250 of 7,985; 3.1%), followed by Bayamón (56 of 18,787; 0.3%), Carolina (nine of 1,865; 0.5%), and Rincón (one of 295; 0.3%). These findings indicated active transmission of DENV-1 (8% of positive pools), DENV-2 (41%), and DENV-3 (51%).

In coordination with PRDH and CDC, PRVCU implemented vector control measures, including wide-area larviciding, mass trapping, adulticidal methods (e.g., space sprays targeting mosquitoes in flight and residual sprays creating long-lasting barriers on surfaces to kill resting mosquitoes), and yard inspections to identify and remove mosquito breeding sites to reduce DENV transmission. In addition, PRDH Environmental Health staff members conducted weekly sanitation surveys, and the U.S. Department of Agriculture provided guidance on appropriate pesticide use in high-risk areas, such as locations with ongoing identification of dengue cases, despite other interventions, and those with significant mosquito insecticide resistance. In September 2024, PRDH established an Integrated Vector Management advisory committee with the purpose of providing vector control recommendations to the Secretary of Health. These recommendations included insecticide rotation and periodic evaluation of insecticides to promote use of effective insecticides and limit emergence of insecticide resistance.

### Public Outreach

By December 2024, response efforts from PRDH, CDC, and PRVCU had reached approximately 160,000 persons through various educational and community engagement initiatives across Puerto Rico. These efforts included 215 activities in priority communities (i.e., areas with high dengue incidence and a history of dengue outbreaks) aimed at raising awareness about dengue prevention, and 328 activities at educational institutions, where dengue-related training and materials were provided. In addition, 1,624 health fairs and community outreach events were held to engage the broader public, and 108 health care facilities were visited to share information on clinical management and prevention strategies. To further support these interventions, 56,809 educational kits were distributed to residents of and visitors to Puerto Rico, equipping participants with valuable resources to aid in dengue prevention efforts. Campaigns such as “*Haz la diferencia y ciérrale la puerta al dengue”* (“Make a difference and close the door to dengue”) were widely promoted across social media^¶^ and radio, encouraging residents to actively participate in removing standing water where mosquitoes can lay eggs, using mosquito repellents to avoid bites, and installing or repairing screens on doors and windows to prevent mosquito entry. 

As part of the public outreach, PRDH and CDC focused on implementation of vaccination with Dengvaxia in populations for whom its use is recommended. Prevaccination dengue screening was established in three clinical laboratory networks. The number of clinics offering the vaccine increased from six to 23 during 2024. However, because of reduced demand for Dengvaxia in the global market, production of the vaccine has been discontinued by the manufacturer, and available doses will expire in 2026. Currently no other dengue vaccines are approved for use in the United States.**

## Discussion

The current dengue outbreak in Puerto Rico is the first since 2013, marking an unusually long (11-year) gap between outbreaks, likely affected by a combination of temporary cross-protective immunity during the Zika outbreak and reduced travel and exposure during the COVID-19 pandemic. The outbreak also began atypically during the low transmission season and intensified into the high season, with case numbers peaking in October and remaining elevated through December 2024. This pattern raises concerns for the potential for high transmission continuing into the next dengue season. The outbreak’s persistence beyond typical seasonal patterns also suggests potential shifts in transmission dynamics, possibly influenced by changes in immunity levels within the population. Persons living in or traveling to Puerto Rico should use Environmental Protection Agency–approved repellents, wear protective clothing, and stay in places with door and window screens. In addition, health care providers should maintain a high suspicion for dengue among persons with fever and potential exposure to mosquitoes in Puerto Rico.

DENV-3, the predominant serotype in this outbreak, accounted for 59.2% of cases with a known serotype, and 58.7% of hospitalizations among cases with known serotype. Its high prevalence might explain the elevated hospitalization rates because DENV-3 is linked to more severe disease (*8*). However, surveillance system changes could also have played a role in the high observed hospitalization rates. For example, to enhance dengue outbreak preparedness, beginning in 2023, PRDH strengthened dengue case management and monitoring in an effort to record more complete information about outcomes. Part of this effort included conducting interviews with all persons suspected to have dengue.

The highest dengue incidence was observed in younger age groups, particularly those aged 10–19 years, highlighting the need for focused outreach and prevention efforts for children and adolescents. In contrast, most fatal cases have occurred among older adults, particularly those aged ≥50 years, underscoring the importance of prioritizing clinical resources for this population. Geographically, although the highest case counts were in the San Juan municipality, the highest dengue incidence occurred in Rincón, Lares, and Maricao, which are all municipalities outside of the metropolitan areas in central and western Puerto Rico.

### Implications for Public Health Practice

The ongoing dengue outbreak underscores the increasing risk of dengue in Puerto Rico and across the Caribbean, Central America, and South America, as the region reported record-breaking case numbers in 2024 (*9*,*10*). The collaborative surveillance and response efforts of PRDH, PRVCU, and CDC, along with targeted outreach in high-risk areas, aimed to equip communities with the knowledge and resources to take proactive measures in preventing dengue transmission. However, more effective tools for dengue control and prevention, including safe and effective dengue vaccines for all age groups and scalable vector control methods, are urgently needed to protect populations at risk in countries with endemic disease and travelers from areas without endemic dengue. Maintaining strong dengue surveillance, improving clinical management, and fostering community awareness will be crucial in mitigating outbreak effects and strengthening public health preparedness and response for future dengue seasons.

## Footnotes

*
https://www.fda.gov/media/124379/download

† http://dx.doi.org/10.15585/mmwr.rr7006a1

§ 45 C.F.R. part 46.102(l)(2), 21 C.F.R. part 56; 42 U.S.C. Sect. 241(d); 5 U.S.C. Sect. 552a; 44 U.S.C. Sect. 3501 et seq.

¶ https://www.salud.pr.gov/dengue; https://www.salud.pr.gov/CMS/146

**
https://www.cdc.gov/dengue/hcp/vaccine/index.html
